# Comparative phylogeography and demographic history of European shads (*Alosa alosa* and *A. fallax*) inferred from mitochondrial DNA

**DOI:** 10.1186/1471-2148-12-194

**Published:** 2012-09-30

**Authors:** Rui Faria, Steven Weiss, Paulo Alexandrino

**Affiliations:** 1 CIBIO, Centro de Investigação em Biodiversidade e Recursos Genéticos, InBIO, Campus Agrário de Vairão, Universidade do Porto, 4485-661, Vairão, Portugal; 2 Departamento de Biologia, Faculdade de Ciências, Universidade do Porto, Rua do Campo Alegre, 4169-007, Porto, Portugal; 3 IBE, Institute of Evolutionary Biology (UPF-CSIC), Departament de Ciències de la Salut i de la Vida, Universitat Pompeu Fabra, PRBB, Doctor Aiguader, 88, 08003, Barcelona, Spain; 4 Karl-Franzens University Graz, Institute of Zoology, Universitätsplatz 2, A-8010, Graz, Austria

## Abstract

**Background:**

Comparative broad-scale phylogeographic studies of aquatic organisms provide insights on biotic responses to the paleohydrological dynamics associated with climatic oscillations. These insights can be used to formulate a framework for understanding the evolutionary history of a species or closely related taxa as well as aid in predictive modeling of further responses to climate change. Anadromous fishes constitute interesting models for understanding the relative importance of environmental versus biological factors in shaping intraspecific genetic substructure on the interface between marine and freshwater realms. European shads, *Alosa alosa* and *A. fallax* are anadromous species that have persisted through historical large-scale environmental perturbations and now additionally face an array of anthropogenic challenges. A comprehensive phylogeographic investigation of these species is needed to provide insights on both the historical processes that have shaped their extant genetic structure and diversity, and the prospects for their future management and conservation.

**Results:**

Despite introgressive hybridization, *A. alosa* and *A. fallax* are genetically divergent, congruent with previous studies. Three similarly divergent mtDNA clades were recognized within both *A. fallax* and *A. alosa*, most likely originating during common periods of isolation during the Pleistocene among the studied oceanographic regions. Periods of basin isolation apparently extended to the Black Sea as additional *Alosa* clades occur there. The present day geographic distribution of genetic diversity within European *Alosa* sp. suggests the existence of a strong but permeable barrier between the Atlantic and Mediterranean seas, as shown for a number of other aquatic species*.* Overall mtDNA diversity is considerably lower for *A. alosa* compared to *A. fallax*, suggesting that the former species is more sensitive to climatic as well as anthropogenic changes. For *A. fallax*, migration from the Mediterranean to the Atlantic was detected but not in the opposite direction, with colonization of the North Atlantic probably occurring after last glacial maximum.

**Conclusion:**

The similar haplotype network topologies between the two species support a common intraspecific history of isolation. Despite these similarities, *A. alosa* and *A. fallax* have clearly responded differently to the hydrological dynamics of the Pleistocene, as reflected in their distinct demographic histories. As the species additionally occupy different ecological niches it should not be surprising that they differ in resilience to natural or human-mediated climatic changes. For *A. fallax*, it is further clear that its demographic response to large-scale hydrological events is not synchronized between the Atlantic and Mediterranean basins. These regional and species-specific differences should be incorporated into future predictive modeling of biological response to climate change as well as current management concepts.

## Background

There is now an extensive literature on the phylogeographic structure of many European freshwater fish species, and these studies often relate to shifting hydrological river networks over several Pleistocene glacial cycles or dating even back into the Pliocene or Miocene epochs [1-7]. Knowledge of broad-scale phylogeographic structure relating to historical geomorphological processes provides us with an evolutionary framework that can support finer-scaled studies directed toward the understanding of speciation processes as well as potential population responses to future climatic changes [8]. Such studies on freshwater systems are now extending into the marine environment, where factors affecting phyogeographic patterns such as ocean currents and larvae dispersal are discussed. Far less attention has been given to the biologically relevant dynamics of the historical landscape for anadromous fishes [9,10], which are invariably more complex as they involve both freshwater and marine environments.

The combined effects of tectonic and climatic processes promotes abrupt sea level changes that alter ocean or sea interconnections, large-scale current patterns, salinity and temperature gradients, as well as water column stratification [11-13]. In Europe such processes can lead to dramatic extinction events such as those experienced in the late Miocene during the Messinian salinity crisis, which resulted in a near drying of the Mediterranean due to volcanic and tectonic uplift near the Strait of Gibraltar [14,15]. More generally, broad-scale isolation mechanisms lead to multi-species phylogeographic breaks such as that found between the Mediterranean and Atlantic basins caused by the Strait of Gibraltar and the complex currents of the Almeria-Oran front. Such breaks have led not only to genetic structure within species but also to levels of reciprocal endemism between these major ocean basins [16,17]. Unlike many purely marine species, whose dispersal is strongly influenced by oceanic currents experienced in the early-life history stages (larvae) [18], dispersal of anadromous species is mainly dependent on adult migration into estuaries and river courses. As such environments in Europe are heavily affected by a wide range of human-related alterations and exploitation pressures [19], the conservation of many anadromous fish populations presents a very difficult challenge.

Shads of genus *Alosa* comprise 16 species that are primarily anadromous representatives of the family Clupeidae, one of the world’s most commercially important fish families [20]. The two European species, *A. alosa* and *A. fallax*, exhibit largely overlapping ranges in the Northeastern Atlantic, which use to extend from Iceland in the north to Morocco in the south ([20,21] and refs. therein). However, human-mediated disturbances (e.g. construction of weirs, water pollution and overfishing) have resulted in a drastic reduction of their range, especially for *A. alosa*, which is now mainly confined to some Iberian and French Atlantic rivers [20,21]. While *A. fallax* was also described in at least one Black Sea tributary and is found throughout the Mediterranean, *A. alosa* is now presumably extinct from the rivers draining into this almost enclosed sea ([20] and refs. therein), though previously reported in Ebro (Spain), Rhone (France) and Moulouya (Morocco). A high diversity of *Alosa* species is found within the Ponto-Caspio region, with three species described for the Black Sea (*A. immaculata*, *A. maeotica* and *A. caspia*); one endemic species (*A. macedonica*) described for Lake Volvi, a Greek freshwater lake; and five species (*A. braschnikowi*, *A. caspia*, *A. kessleri, A. saposchnikowii* and *A. sphaerocephala*) described for the Caspian Sea. Thus, these species occur across oceanic biogeographic regions where abiotic factors such as currents, salinity and temperature continuously change allowing episodes of long-distance dispersal, gene flow and alternatively isolation (e.g. [16,22-24]).

Despite their biological interest as a model to study the main factors involved in diversification in the region, their phylogenetic relationships have not yet been completely clarified [25,26] and little is known about the geographic distribution of their genetic variability [27-30]. In particular, given the limited number of samples analyzed per species in phylogenetic studies [25,26], it is not clear if the monophyletic status of European shad species will be maintained with increased geographic sampling coverage. Moreover, despite the identification of significant genetic differentiation between populations, mainly among *A. fallax* populations, some studies have a very regional focus [31,32], while others are based on genetic markers with limited variability for drawing broad geographic-scale conclusions (e.g. [28,30]), highlighting the need for a comprehensive phylogeographic study.

Hybridization among European shad species is well documented ([20,21] and refs. therein), with levels of introgression varying widely among the populations thus far investigated [28-32]. Nonetheless, in all locations where species are found in sympatry, taxa remain largely distinct reflecting a degree of resilience to the formation of hybrid swarms and partial barriers to introgression. Understanding cladogenesis, ecological divergence and varying degrees of reproductive isolation despite gene flow (i.e. introgression) remains a major challenge for evolutionary geneticists and thus shads in the Atlantic-Mediterranean region provide a unique model for population genetic studies of these processes.

In order to obtain a better understanding of the roles of historical events and demographic processes in shaping genetic diversity of European *Alosa* we carried out the first comprehensive comparative phylogeographic analysis of these species. We were particularly interested in: i) re-evaluating the genetic relationships among *Alosa* species; ii) testing if shared mtDNA haplotypes between *A. alosa* and *A. fallax* result from introgression or incomplete lineage sorting; iii) understanding how genetic variability is distributed among the Eastern Atlantic and Mediterranean basins, as well as inferring the major phylogeographic barriers to gene flow that may have been responsible for promoting differentiation; and, iv) understanding the impact of major Pleistocene climatic changes on the genetic composition and historical demography of *A. alosa* and *A. fallax* populations from different geographic regions. This study focuses on historical patterns and processes, but the inferences can contribute to the improvement of conservation measures in the future, as the species confront both natural and man-made ecosystem alterations.

## Results

### DNA sequence variation, phylogenetic relationships and divergence time between Eurasian shads

The final alignment yielded 448 bp for *cyt b* (*N* = 335) and 975 bp for *ND1* (*N* = 273), with no indels or stop codons, after removing the terminal stop codon for *ND1*. In 11 *A. fallax* individuals, more than one haplotype was observed within a single individual, corresponding to common haplotypes in our database. As the occurrence of heteroplasmy was reported previously in *Alosa*[33], for simplicity, we excluded this low percentage (about 3%) of putative heteroplasmic individuals from further analyses.

**Figure 1:**
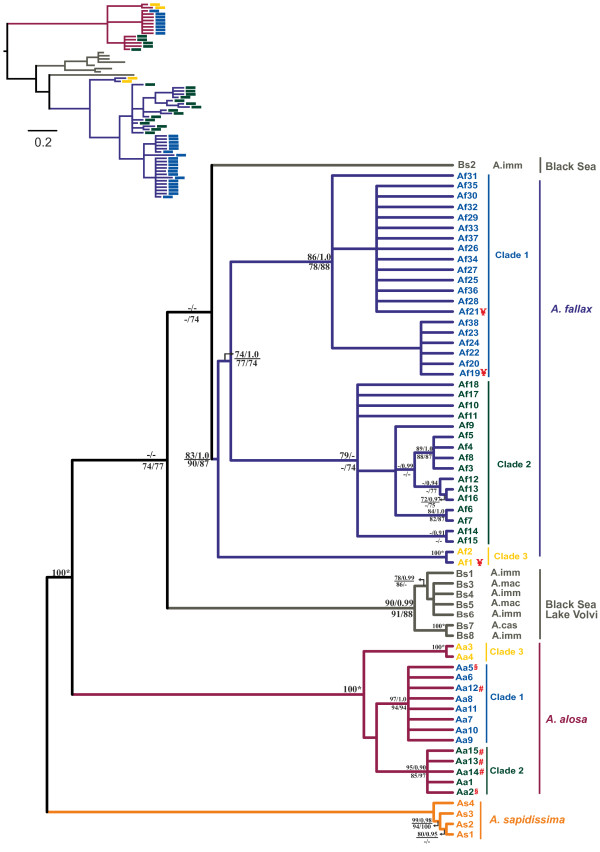
**Majority rule consensus parsimony tree based on two concatenated mtDNA genes (*ND1 *and *cyt b*).** For the major clades of the main tree, values equal to or over 70% are shown for ML (above, left); MP (below, left); and NJ (below, right); and above 0.90 for B (above, right). 100* means that all bootstrap values and posterior probabilities are equal to or higher than 95. The tree was rooted with *A. sapidissima*. The lineages corresponding to the main taxonomic units analyzed were colored differently. *A. alosa* and *A. fallax* haplotype codes are also presented in three distinct colors, representing the main clades found within each species, as displayed in Figure 2. Three classes of introgressed haplotypes were found between *A. alosa* and *A. fallax*: ♯, haplotypes only found in phenotypically designated *A. fallax* but located in the *A. alosa* clade; §, haplotypes found in phenotypically designated *A. alosa* and *A. fallax,* but located in the *A. alosa* clade; and ¥, haplotypes found in phenotypically designated *A. alosa* and *A. fallax* but found in the *A. fallax* clade. A Bayesian tree is presented in the left upper corner for branch length visualization. Colors correspond to those shown in the main tree. Although *A. sapidissima* was also used as an outgroup in the construction of the Bayesian tree, this was later removed from the figure for simplification.

**Figure 2:**
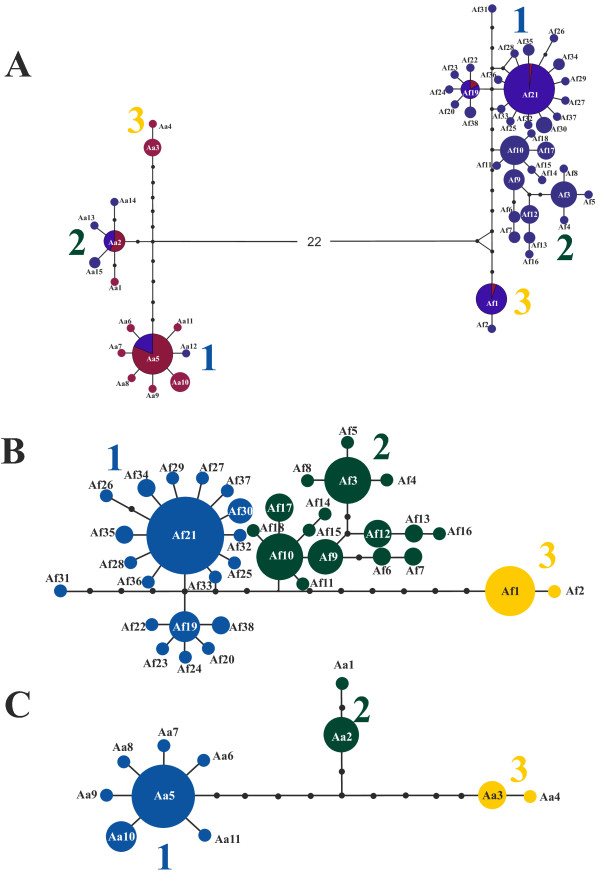
**Median-Joining (MJ) and Reduced Median (RM) haplotype networks for both genes concatenated (*ND1 *and *cyt b*).A**) Including all individuals analyzed in this study. Haplotypes found in individuals classified morphologically as *A. fallax* are represented in purple, while haplotypes found in individuals classified morphologically as *A. alosa* are colored in dark red. Shared haplotypes are represented by pie charts with the proportions reflecting the relative frequency of those haplotypes in *A. alosa* (dark red) and *A. fallax* (purple). The number (22) shown between the two main lineages (*A. alosa* and *A. fallax*) represents the number of substitutions. **B**) Haplotypes found in the 29 populations of *A. fallax* analyzed, excluding putative introgressed individuals; **C**) Haplotypes found in the nine populations of *A. alosa* analyzed*,* excluding putative introgressed individuals. In figures B and C, each clade is represented by different colors to facilitate the comparison with Figures 1 and 3. The area of each circle is proportional to the haplotype frequency. Each black dot represents a missing haplotype. Haplotype and clade nomenclatures correspond to those used in Figure 2 and Tables 4 and 5.

There were a total of 127 variable positions, 92 of which were parsimony informative defining a total of 65 haplotypes: 11 found in *A. alosa* and 42 in *A. fallax* individuals, from which 4 are shared among the two species. Maximum parsimony (MP) analysis revealed 60 equally parsimonious trees of 177 steps (CI = 0.7282; RI = 0.9484). The best-fit model was TrN + I + G with an estimate of invariable sites (I = 0.6479) and a discrete approximation of the gamma distribution (alpha = 0.8521). As similar topologies were obtained with all methods only the MP and Bayesian trees are shown (Figure 1). The monophyly of all Eurasian *Alosa* is very well supported by the mtDNA fragments here analyzed. Notwithstanding the observation of shared haplotypes between *A. alosa* and *A. fallax* (Figures 1 and 2A), a very well supported clade is formed by *A. alosa* haplotypes, while *A. fallax* and Black Sea species complex (BSC) taxa form a large diverse clade with low support (Figure 1), as previously shown [25]. Excluding introgressed individuals, three subgroups of *A. alosa* haplotypes each form well-supported clades, while the support for three subclades of *A. fallax* haplotypes and the primary clade (excluding the Bs2 haplotype) of BSC was generally lower, except for the Bayesian analysis, where posterior probability is generally equal to 1.0 (Figure 1). Divergence time between *A. alosa* and other major clades of Eurasian shads (BSC and *A. fallax*) ranges from 0.85 to 1.25 Myrs (assuming 2% divergence rate), being lower between *A. fallax* and the BSC (0.45-0.75) (Table 1). Two clades are observed within the Black Sea (excluding Bs2) (Figure 1). Within both *A. alosa* and *A. fallax*, the three main well-supported clades started to diverge between 0.25 and 0.40 Myrs ago; and between 0.20 and 0.45 Myrs ago, respectively (Table 1). Importantly, intraspecific divergence (within *A. alosa* and *A. fallax*) is always lower than interspecific divergence (among *A. alosa*, *A. fallax* and BSC).

**Table 1:** **Estimates of divergence among the three main lineages of Eurasian shads observed in Figure**1**(*A. alosa*, *A. fallax *and Black Sea species complex), and among the three main clades identified within *A. alosa *and *A. fallax *using the two genes combined (*ND1 *and *cyt b*)**

	**Divergence**		**Divergence time (Myrs) 1%**	**Divergence time (Myrs) 2%**	**Divergence time (Myrs) 4%**
	***p *distance**				
	**D**_***a***_	**D**_***xy***_			
*A. alosa*/*A. fallax*	0.020	0.025	2.000-2.500	1.000-1.250	0.500-0.625
*A. alosa*/Black Sea	0.017	0.023	1.700-2.300	0.850-1.150	0.425-0.575
*A. fallax*/Black Sea	0.009	0.015	0.900-1.500	0.450-0.750	0.225-0.375
*A. alosa*					
clade1/clade2	0.005	0.006	0.500-0.600	0.250-0.300	0.125-0.150
clade1/clade3	0.007	0.008	0.700-0.800	0.350-0.400	0.175-0.200
clade2/clade3	0.005	0.006	0.500-0.600	0.250-0.300	0.125-0.150
*A. fallax*					
clade1/clade2	0.004	0.006	0.400-0.600	0.200-0.300	0.100-0.150
clade1/clade3	0.007	0.009	0.700-0.900	0.350-0.450	0.175-0.225
clade2/clade3	0.006	0.008	0.600-0.800	0.300-0.400	0.150-0.200

D_*xy*_, mean pairwise divergence between groups; D_*a*_, net nucleotide divergence between groups; Black Sea represents all haplotypes found in the BSC complex, including Bs2. Estimates using different divergence rates (1-4%) are presented.

The posterior distribution of the divergence time between *A. alosa* and *A. fallax* obtained with IMa2 did not show a well-defined peak, suggesting that there is not enough information in the data to confidently infer this parameter (Additional file 1: Figure S1A). Nonetheless, the bins with higher posterior probabilities correspond to a divergence time of around 1.26 Myrs (Table 2), which is very close to the estimate based on the amount of sequence divergence (1.00-1.25 Myrs; Table 1).

**Table 2:** **Maximum likelihood estimates of peak posterior distribution and 95% highest posterior densities (HPD) of effective population sizes (Ne), effective number of migrants per generation in each direction (2Nm) and time since divergence (t) in million years (Myrs) between *A. alosa *and *A. fallax *and between two geographic groups of *A. fallax *, as inferred with IMa2**

**Species/Group**	**Ancestral Ne [95%HPD]**	**Ne of descendant populations [95%HPD]**	**2 Nm [95%HPD]**	**(t) [95%HPD]**
*A. alosa*/*A. fallax*	189,532	*A. alosa*: 63,303	Into *A. alosa*: 2.630***	1.259
	[28,154–386,803]^3^	[29,692–122,514]	[0.486–8.558]	[0.294–3.477]^4^
		*A. fallax*: 228,909	Into *A. fallax*: 2.300*	
		[158,862–322,562]	[0–9.647]	
Mediterranean/Atlantic^1^	-	Mediterranean: 91,749	Into Mediterranean:0.008^ns^	0.332
	[0–348,127]^3^	[48,560–166,117]	[0–2.370]	[0.153–1.496]^4^
		Atlantic: 144,733	Into Atlantic: 2.149***	
		[87,008–231,952]	[0.299–6.932]	
Mediterranean/Atlantic^2^	30,830	Mediterranean: 88,180	Into Mediterranean: 0.008^ns^	0.250
	[0–348,094]^3^	[45,929–163,432]	[0–2.538]^3^	[0.116–1.494]^4^
		Atlantic: 126,331	Into Atlantic: 1.905**	
		[72,940–212,094]	[0.248–6.774]	

^1^all sequences included; ^2^excluding sequences putatively originated from a third group (Morocco); ^3^ non-informative HPD because the posterior density does not reach low levels near neither the upper nor the lower limit of the prior; ^4^HPD interval may be incorrect due to multiple peaks; ^*^Values significantly different from zero at p < 0.05; **p < 0.01; and ***p < 0.001; ^ns^non significant.

### Introgressive hybridization between *A. alosa* and *A. fallax*

In three out of nine *A. alosa* populations analyzed, we found *A. fallax* haplotypes, and in 12 of the 29 populations of *A. fallax* we found varying percentages of *A. alosa* haplotypes, with relatively high levels (25-63%) in populations from the United Kingdom (Usk and Tywi) and Portugal (Lima and Tejo) (Table 3, Figure 2A). Coalescent-based IMa2 analysis detected statistically significant gene flow between the two species in both directions (Table 2; Additional file 1: Figure S1C). Although effective migration is slightly higher into *A. alosa* than into *A. fallax*, the confidence intervals overlap considerably (Table 2).

**Table 3:** **Percentage of mtDNA haplotypes (*cyt b*) resulting from hybridization and introgression between *A. alosa *and *A. fallax***

	**Populations**	***N***	**Number of*A. alosa*mtDNA haplotypes**	**Number of*A. fall*ax mtDNA haplotypes**
*A. alosa*				
	Garonne	9	8	1 (11%)
	Dordogne	11	10	1 (9%)
	Guadiana	9	8	1 (11%)
*A. fallax*				
	Scotland	8	1 (13%)	7
	Severn*	18	4 (22%)	14
	Wye	14	3 (21%)	11
	Usk	11	5 (46%)	6
	Tywi	16	10 (63%)	6
	Minho	7	1 (14%)	6
	Lima	16	4 (25%)	12
	Mondego	11	2 (18%)	9
	Tejo	8	2 (25%)	6
	Guadiana	7	1 (14%)	6
	Herault	5	1 (20%)	4
	Rhône	7	1 (14%)	6

In the remaining populations no introgression was observed.

*individuals from Severn and its tributary, the Teme, were pooled.

### Intraspecific phylogeographic network description

**Figure 3:**
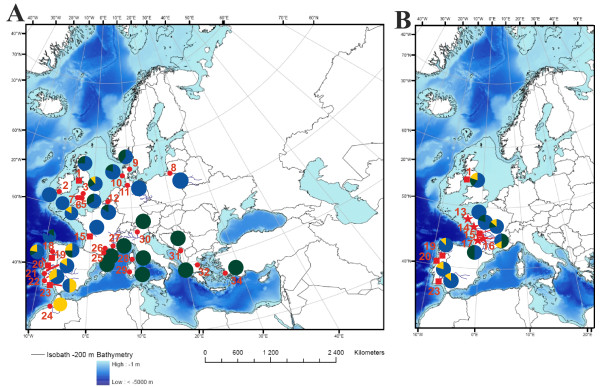
**Maps representing the geographic distribution of the main haplogroups.A**) Frequency of the *A. fallax* haplogroups found in 29 populations of *A. fallax*; and **B**) Frequency of the *A. alosa* haplogroups found in nine populations of *A. alosa*. Numbers correspond to the populations represented in Figure 1 and Tables 4 and 5. Haplotypes resulting from introgression are not shown. Pie charts represent the relative frequency of the main haplogroups found in each sample location.

For *A. fallax*, the 38 haplotypes across all three clades span a total of 15 substitutions (Figure 2B, Table 4). One group of haplotypes (clade 1) is distributed throughout the Atlantic north of Morocco (Figures 2B and 3A). Within this group, a large star-like cluster of haplotypes is centered on the most common haplotype (Af21) found throughout the Atlantic, north of the Mira River in Portugal (Figure 2B, Table 4). A second small star-like cluster of haplotypes is closely related to the large cluster, representing haplotypes common to Portuguese rivers. A second large diverse group of haplotypes (clade 2: Af3-Af18) is a minimum of four substitutions divergent from all other haplotypes and represents populations distributed throughout the Mediterranean basin, although the two most common haplotypes (Af3 and Af10) were also found in Atlantic populations, with Af3 found as far north as Denmark (Figures 2B and 3A). Two additional haplotypes (Af1 and Af2: clade 3) are a minimum of eight substitutions divergent from all others. One of these haplotypes is fixed in Morocco and widely distributed in Portugal (Af1), while the other (Af2) was only detected in the Guadiana River. An additional divergent haplotype (Af31) was found in the Tejo River, Portugal (Figure 2B, Table 4). Some haplotypes were found in a single river or confined to a restricted geographic region (e.g. Af30 in Severn, Atlantic; e.g. Af9 in Greece and Turkey, Mediterranean) (Table 4).

**Table 4:** **Geographical distribution and frequency of mtDNA haplotypes (both genes combined) found in *A. fallax *populations after excluding those that cluster within the *A. alosa *haplogroup (putatively resulting from introgression from *A. alosa*)**

	***N***	**Af 1**	**Af2**	**Af3**	**Af4**	**Af5**	**Af6**	**Af7**	**Af8**	**Af9**	**Af10**	**Af11**	**Af12**	**Af13**	**Af14**	**Af15**	**Af16**	**Af17**	**Af18**	**Af19**	**Af20**	**Af21**	**Af22**	**Af23**	**Af24**	**Af25**	**Af26**	**Af27**	**Af 28**	**Af29**	**Af30**	**Af31**	**Af32**	**Af33**	**Af34**	**Af35**	**Af36**	**Af37**	**Af38**
Curonian (8)	5	-	-	-	-	-	-	-	-	-	-	-	-	-	-	-	-	-	-	-	-	5	-	-	-	-	-	-	-	-	-	-	-	-	-	-	-	-	-
Albaek (9)	5	-	-	2	-	-	-	-	-	-	-	-	-	-	-	-	-	-	-	-	-	3	-	-	-	-	-	-	-	-	-	-	-	-	-	-	-	-	-
Wadden (10)	5	-	-	1	-	-	-	-	-	-	-	-	-	-	-	-	-	-	-	-	-	3	-	-	-	-	-	-	-	-	-	-	-	-	-	-	1	-	-
Elbe (11)	5	-	-	-	-	-	-	-	-	-	-	-	-	-	-	-	-	-	-	-	-	4	-	-	-	-	-	-	-	-	-	-	-	-	1	-	-	-	-
Schedlt (12)	6	-	-	1	-	-	-	-	-	-	-	-	-	-	-	-	-	-	-	-	-	3	-	-	-	1	-	-	-	-	-	-	-	-	1	-	-	-	-
Scotland (1)	4	-	-	-	-	1	-	-	-	-	-	-	-	-	-	-	-	-	-	-	-	2	-	-	-	-	-	-	-	1	-	-	-	-	-	-	-	-	-
Severn (3)	9	1	-	2	-	-	-	-	-	-	-	-	-	-	-	-	-	-	-	-	-	3	-	-	-	-	-	-	-	-	3	-	-	-	-	-	-	-	-
Wye (5)	6	-	-	2	-	-	-	-	-	-	-	-	-	-	-	-	-	-	-	-	-	3	-	-	-	-	1	-	-	-	-	-	-	-	-	-	-	-	-
Usk (6)	6	1	-	-	-	-	-	-	-	-	-	-	-	-	-	-	-	-	-	-	-	4	-	-	-	-	-	-	1	-	-	-	-	-	-	-	-	-	-
Tywi (7)	4	-	-	-	-	-	-	-	-	-	-	-	-	-	-	-	-	-	-	-	-	2	-	-	-	-	-	-	-	-	-	-	-	-	-	2	-	-	-
Leane (2)	5	-	-	-	-	-	-	-	-	-	-	-	-	-	-	-	-	-	-	-	-	4	-	-	-	-	-	-	-	-	-	-	1	-	-	-	-	-	-
Charente (15)	5	-	-	-	-	-	-	-	-	-	-	-	-	-	-	-	-	-	-	-	-	3	-	-	-	-	-	1	-	-	-	-	-	-	-	-	-	1	-
Minho (18)	6	-	-	-	-	-	-	-	-	-	1	-	-	-	-	-	-	-	-	1	-	4	-	-	-	-	-	-	-	-	-	-	-	-	-	-	-	-	-
Lima (19)	8	2	-	-	-	-	-	-	-	-	2	-	-	-	-	-	-	-	-	-	-	3	1	-	-	-	-	-	-	-	-	-	-	-	-	-	-	-	-
Mondego (20)	9	2	-	-	-	-	-	-	-	-	1	-	-	-	-	-	-	-	-	-	1	5	-	-	-	-	-	-	-	-	-	-	-	-	-	-	-	-	-
Tejo (21)	7	1	-	-	-	-	-	-	-	-	-	-	-	-	-	-	-	-	-	2	-	1	-	1	-	-	-	-	-	-	-	1	-	1	-	-	-	-	-
Mira (22)	3	1	-	-	-	-	-	-	-	-	-	-	-	-	-	-	-	-	-	-	-	-	-	-	-	-	-	-	-	-	-	-	-	-	-	-	-	-	2
Guadiana (23)	6	2	1	-	-	-	-	-	-	-	-	-	-	-	-	-	-	-	-	2	-	-	-	-	1	-	-	-	-	-	-	-	-	-	-	-	-	-	-
Sebou (24)	8	8	-	-	-	-	-	-	-	-	-	-	-	-	-	-	-	-	-	-	-	-	-	-	-	-	-	-	-	-	-	-	-	-	-	-	-	-	-
Aude (25)	5	-	-	1	1	-	-	-	-	-	3	-	-	-	-	-	-	-	-	-	-	-	-	-	-	-	-	-	-	-	-	-	-	-	-	-	-	-	-
Herault (26)	4	-	-	1	-	-	-	-	-	-	3	-	-	-	-	-	-	-	-	-	-	-	-	-	-	-	-	-	-	-	-	-	-	-	-	-	-	-	-
Rhone (27)	5	-	-	2	-	-	-	-	1	-	1	-	-	-	-	-	-	-	1	-	-	-	-	-	-	-	-	-	-	-	-	-	-	-	-	-	-	-	-
Corsica (28)	6	-	-	-	-	-	-	-	-	-	-	-	4	-	-	-	-	2	-	-	-	-	-	-	-	-	-	-	-	-	-	-	-	-	-	-	-	-	-
Sardinia (29)	4	-	-	-	-	-	-	-	-	-	-	-	1	-	-	-	-	3	-	-	-	-	-	-	-	-	-	-	-	-	-	-	-	-	-	-	-	-	-
Garda (30)	6	-	-	-	-	-	-	-	-	-	4	-	-	-	1	1	-	-	-	-	-	-	-	-	-	-	-	-	-	-	-	-	-	-	-	-	-	-	-
Skadar (31)	5	-	-		-	-	-	-	-	-	-	1	1	2	-	-	1	-	-	-	-	-	-	-	-	-	-	-	-	-	-	-	-	-	-	-	-	-	-
Pinios (32)	7	-	-	-	-	-	-	-	-	7	-	-	-	-	-	-	-	-	-	-	-	-	-	-	-	-	-	-	-	-	-	-	-	-	-	-	-	-	-
Izmir (34)	5	-	-	-	-	-	2	2	-	1	-	-	-	-	-	-	-	-	-	-	-	-	-	-	-	-	-	-	-	-	-	-	-	-	-	-	-	-	-

Codes for populations and haplotypes are the same as in Table 8, as well as in the different figures.

For *A. alosa*, 11 haplotypes across the three clades span a maximum of 12 substitutions whereby one star-like cluster (clade 1) centers on the most common and widespread haplotype (Aa5) found in nearly all populations (Figures 2C and 3B; Table 5). Clade 1 is separated by a minimum of seven substitutions from the four remaining haplotypes, which are dispersed over two groups, one mainly located in the northern Atlantic (Aa1 and Aa2; clade 2), and the second (clade 3) represented by primarily low-frequency haplotypes with no apparent phylogeographic pattern (Figures 2C and 3B). The Guadiana River was fixed for a unique haplotype, while Lima River exhibited a total of six haplotypes whereas four of them were found in single individuals (i.e. singletons) (Table 5).

**Table 5:** **Geographical distribution and frequency of mtDNA haplotypes (both genes combined) found in *A. alosa*populations after excluding those that cluster within the *A. fallax *haplogroup (putatively resulting from introgression with *A. fallax*)**

	***N***	**Aa1**	**Aa2**	**Aa3**	**Aa4**	**Aa5**	**Aa6**	**Aa7**	**Aa8**	**Aa9**	**Aa10**	**Aa11**
Scotland (1)	5	-	1	1	-	3	-	-	-	-	-	-
Aulne (13)	4	-	-	-	-	4	-	-	-	-	-	-
Vienne (14)	5	-	1	-	-	4	-	-	-	-	-	-
Charente (15)	7	-	1	-	1	5	-	-	-	-	-	-
Dordogne (16)	6	1	2	1	-	2	-	-	-	-	-	-
Garonne (17)	6	-	3	-	-	2	-	-	-	-	-	1
Lima (19)	7	-	-	1	-	2	1	1	1	1	-	-
Mondego (20)	5	-	-	1	-	4	-	-	-	-	-	-
Guadiana (23)	7	-	-	1	-	-	-	-	-	-	6	-

Codes for populations and haplotypes are the same as in Table 8, as well as in the different figures.

### Population substructure

Within population variation explained the majority of the genetic variance in both European shad species (ca. 55% in *A. fallax*, and 96% in *A. alosa*; 2-level AMOVA) (Table 6). Nonetheless, among population variance was significant in *A. fallax* (Φ_ST_ = 0.44961; p < 0.0001) but not in *A. alosa* (Table 6). Levels of differentiation within the Mediterranean basin for *A. fallax* were higher than in the Atlantic (2-level AMOVA results; Φ_ST_=0.38730 and Φ_ST_=0.27173, respectively; Table 6). For *A. fallax* the 3-level AMOVA revealed significant structure between the Atlantic, Morocco and Mediterranean population groups (51.52%; Φ_CT_ = 0.51524; p < 0.0001). A SAMOVA considering *k* = 3 showed that this clustering indeed maximized Φ_CT_ (0.51524), supporting our grouping for the AMOVA structure analysis.

**Table 6:** **Analysis of molecular variance (AMOVA) in *A. alosa *and *A. fallax***

	**Source of variation**	**df**	***p*value**	**Variance components**	**% Variation**	**Ф-statistics**
*A. alosa*						
2-level	All populations (AP)	8	0.22	0.09141	4.30	*Φ*_ST_ = 0.04301
2-level	Northern Atlantic (AP)	5	0.4	0.00028	0.01	*Φ*_ST_ =0.00013
2-level	Southern Atlantic (AP)	2	0.35	0.00405	0.22	*Φ*_ST_ =0.00220
*A. fallax*						
2-level	All populations (AP)	27	< 0.0001	1.32962	44.96	*Φ*_ST_ =0.44961
2-level	Atlantic (AP)	18	< 0.0001	0.72105	27.17	*Φ*_ST_ =0.27173
2-level	Mediterranean (AP)	8	< 0.0001	0.55727	38.73	*Φ*_ST_ =0.38730
3-level	Among groups (AG)*	2	< 0.0001	2.05054	51.52	*Φ*_CT_ =0.51524
	Among populations within groups (APWG)	25	< 0.0001	0.30157	7.58	*Φ*_SC_ =0.15632
	Within populations (WP)	131	< 0.0001	1.62767	40.90	*Φ*_ST_ =0.59102

* Populations were clustered in three main groups: Atlantic (except Morocco); Mediterranean; and Morocco. *AP*, among populations; *AG*, among groups; *APWG*, among populations within groups; *WP*, within populations.

Divergence time between the Atlantic and Mediterranean groups of populations estimated based on an isolation-with-migration model dates to 0.33 Myrs ago (0.25 if we exclude the haplotypes from clade 3). Although a 95% HPD upper bound could not be computed for the coalescent-based estimates of divergence time (Table 2; Additional file 1: Figure S1D), the values obtained are very close to the interval of divergence time based on sequence divergence (0.20 to 0.30 Myrs) (Table 1). Shared haplotypes between Atlantic and Mediterranean populations were observed, as well as between Atlantic populations and Morocco (Table 4). Coalescent-based estimates of migration between the former pair of populations identified significant migration from Mediterranean into the Atlantic coasts of Europe but not the reverse (not significantly different from zero) (Table 2; Additional file 1: Figure S1F).

### Intraspecific variation and demographic history

Both haplotype and nucleotide diversities, as well as observed theta are higher for *A. fallax* (*h* = 0.861; π = 0.00432; θ = 8.861) compared to *A. alosa* (*h* = 0.715; π = 0.00304; θ = 4.647) (Table 7). Accordingly, the coalescent-based IMa2 estimate of the effective population size of *A. alosa* is considerably lower than observed for *A. fallax* (around 63,000 and 229,000 individuals, respectively) with non-overlapping confidence intervals (Table 2; Additional file 1: Figure S1B). The posterior distribution of the ancestral population size is very flat, with a very wide likelihood-based confidence interval.

**Table 7:** Summary statistics and demographic parameters based on the combined genes for different data subsets

		**(*h*)**	**(π) (%)**	**Theta**	**Tajima’s*D***	***R***^**2**^	***F***_**s**_	**Theta**_**0**_	**Tau (τ)**	**TSE (1%)**	**TSE (2%)**	**TSE (4%)**
*A. alosa* (n = 52)		0.715	0.304	4.647								
	Clade 1 (n = 37)	0.489	0.039	1.437	−1.677	0.0615	−4.762**	0	0.550	38,650	19,325	9,663
	Clade 2 (n = 9)	0.222	0.031	0.736	−1.362	0.3143	0.671					
	Clade 3 (n = 6)	0.333	0.023	0.438	−0.933	0.3727	−0.003					
*A. fallax* (n = 159)		0.861	0.432	8.861								
	Clade 1 (n = 80)	0.575	0.086	4.644	−2.314**	0.0301**	−18.820***	0.797	0.372	26,142	13,071	6,543
	Clade 2 (n = 60)	0.872	0.209	3.860	−0.786	0.0753	−4.641*	0.615	2.265	159,170	79,585	39,793
	Clade 3 (n = 19)	0.105	0.008	0.286	−1.1640	0.2233	−0.838					

*p < 0.05; **p < 0.01; ***p < 0.001.Shown is (n), number of individuals; (*h*), haplotype diversity, (π), nucleotide diversity; (τ), mutational time since demographic expansion; (theta) the mutation parameter per sequence as observed and (theta_0_) prior to expansion; TSE, Time since the last population expansion; (Tajima’s *D*), Tajima’s statistics (1989); (*R*^2^), Ramos-Onsins and Rozas statistics (2002); (*F*_s_), Fu’s statistics (1997); (the estimated time since expansion is only given for those clades/taxonomic units with significant population growth identified by more than one method (Tajimas’s *D*, *R*^2^, or *F*_s_), using different divergence rates.

Within *A. fallax*, clade 2 (predominantly with a Mediterranean distribution) showed higher levels of both nucleotide and haplotype diversity compared to clade 1 (Atlantic populations, except Sebou) (Table 7). However, both theta and the coalescent-based IMa2 estimates of the effective population size of clade 1 are considerably higher than observed for clade 2 (around 126,000 and 88,000 individuals, respectively, but with largely overlapping confidence intervals) (Table 3; Additional file 1: Figure S1E). Within the Atlantic basin, both *A. fallax* and *A. alosa* revealed higher diversity levels in the Iberian Peninsula region compared to the North Atlantic (data not shown). Within *A. fallax*, a demographic expansion was supported for clade 1 (primarily in the Atlantic basin) and clade 2 (primarily in the Mediterranean basin) starting about 13,000 and 80,000 yrs ago, respectively (Table 7). Within *A. alosa* clades, growth was supported only for clade 1, beginning about 19,000 yrs ago (Table 7). Despite the large confidence intervals, the Bayesian skyride analysis confirms a general tendency for population growth for these same clades during the last 60,000 years, with exception of the last thousand years. Although the time since expansion estimates based on the Bayesian skyride and on the one-time sudden exponential growth model analyses (see methods) are not completely coincident, we observe a similar trend: in *A. fallax*, effective population size started to increase earlier in clade 2 (primarily the Mediterranean basin) than in clade 1 (Atlantic basin).

## Discussion

### Eurasian shad diversification

Our analysis provides strong support for the monophyly of Eurasian shads yet weak support for the internal branching order of major European lineages, as previously shown with a far more limited data set [25]. The lack of resolution for the relationships among major lineages presumably reflects relatively rapid radiation during the Pleistocene (i.e. a hard polytomy) but whole mtDNA genome data or an extensive nuclear gene data set might still provide more resolution. Even the question of the sister species status of *A. alosa* and *A. fallax*[25,28] cannot be unequivocally answered due to the very weak node support of the clade grouping *A. fallax* and BSC haplotypes (Figure 1). However, divergence estimates (Table 1) favor a considerably closer relationship of *A. fallax* to the BSC than to *A. alosa*.

For estimated divergence times among lineages we rely on a credible but rough divergence rate of 2% /Myrs for the whole mtDNA, though we provide inferences based on a range from 1-4%/Myrs (Table 1). Rates such as 1%/Myrs are very unlikely as this would result in estimates of demographic expansion in the Atlantic (Clade 1 in both *A. alosa* and *A. fallax*) ca. 25–40 thousand years ago, which is at the height of the last glacial maximum (LGM) (see Table 7). Rates of 3%/Myrs have been convincingly shown for the genus *Coregonus*[34] whereby their calibration is based on a more recent split, and there is mounting evidence that there is a time-decay phenomenon for mtDNA divergence rates due to the delayed efficiency of purifying selection within a phylogeny [35,36]. Moreover, there is little evidence for considerably higher rates of divergence for temperate freshwater fishes (see discussion on cold-tolerant fishes in [7,37]). Thus, we limit our discussions to inferences based on a 2%/Myrs divergence rate, but emphasize that the relative as opposed to the absolute dating of events among lineages is more credible.

Using this framework, some general inferences can be drawn concerning the diversification of European *Alosa* lineages. First, all species or clade splits are confined to the Pleistocene implying that these cold-tolerant taxa proliferated or radiated during glacier-mediated climatic oscillations and not during pre-Pleistocene (i.e. Pliocene) climatic conditions, which were considerably warmer [38], or perhaps even unsuitable for *Alosa* in the region under study. Second, all species or clade splits occurred over time periods older than the LGM and thus this most recent glacial cycle had little to nothing to do with the origin of major lineages. The divergence between *A. fallax* and the Black Sea lineages started roughly 0.45 to 0.75 Myrs ago. We speculate that a radiation occurred during this period when connections between the Mediterranean and Black seas (as well as Caspian) took place [39], allowing the eastern expansion of *Alosa* into the Black and Caspian seas. These connections were subsequently interrupted several times (see [22] and refs. therein, [39]), promoting isolation and differentiation, providing a clearer biogeographic break, which has presumably played a role in promoting cladogenesis [40]. Similar to the communication between the Mediterranean and Atlantic basins, however, there is presently no absolute barrier between the Mediterranean and Black seas. The present connection occurs over a salinity gradient along the Dardanelles channel, which connects the Aegean and Marmara seas and has bottom salinities similar to the Mediterranean. The Marmara Sea is then connected to the Black Sea via the Bosporus strait, whose total saline input into the Black Sea combined with the present freshwater inputs results in an approximately 17.5 to 19 ppt surface water salinity [23]. This raises the question of whether the observed cladogenesis and speciation in the genus *Alosa* in this region is primarily promoted through allopatry stemming from phases of geographic isolation during reduced Mediterranean Sea levels, or simply the starkly differing environmental conditions (or both), as suggested for other species in the Ponto-Caspio region [22].

Salinity gradients or breaks have clearly played a role in the diversification of the genus *Alosa.* Several landlocked populations of *Alosa* complete their entire life cycle in freshwater and differ in terms of morphology, genetics and physiology from nearby anadromous populations [20,27,41,42]. *A. macedonica*, endemic to freshwater Lake Volvi in Greece, is one such example. Freshwater lakes of this region were presumably colonized with *Alosa* immigrating from a low salinity phase of the Black Sea during one of its spills into the Aegean Sea [43]. *Alosa* colonized two lakes on the Greek peninsula (Lake Volvi and Lake Vistonis), whereby the Lake Vistonis lineage has apparently gone extinct due perhaps to human-caused salt water intrusion [43]. *Alosa* from Lake Volvi is considered a distinct species carrying out its entire life cycle in freshwater, whereas the Aegean Sea region, now with higher salinity harbors only *A. fallax* of Mediterranean origin.

### Interspecific gene flow versus ancestral polymorphism

While ancestral polymorphism could in theory explain shared haplotypes between the two species, the evidence for hybridization and introgression is overwhelming, in agreement with previous published studies [20,27,28]. The occurrence of apparent hybrids is not geographically homogeneous, with certain rivers showing high and others low frequencies. This alone is suggestive of hybridization in contrast to ancestral polymorphism, which should show little geographic pattern. However, given the anadromy and natal site fidelity of the species, such observations are not 100% conclusive.

Coalescent-based estimates under a model of isolation-with-migration support gene flow between *A. alosa* and *A. fallax*. Strong population substructure or complex demographic scenarios may provide violations of model assumptions. However, several lines of argument strongly support that introgression is occurring between the two species. First, it has been shown that migration estimates based on isolation-with-migration models are robust to population structure and departures from simple demographic scenarios [44]. Second, the occurrence of morphological hybrids between these species has been widely documented [20]. And finally, previous studies showed a correlation between genotypes based on nuclear markers and gill raker counts, with the hybrids presenting intermediate morphological and genetic compositions, strongly supporting the occurrence of hybridization [28].

Interestingly, the levels of introgression are particularly high in *A. fallax* populations of Usk and Tywi (Wales, UK), although *A. alosa* basically disappeared from this region [27,32]. These results are concordant with the higher mtDNA introgression levels previously found in these same rivers [27]. The interpretation of these results under a wider geographic perspective suggests that hybridization between *A. alosa* and *A. fallax* varies among river basins from 0 to 63%, reaching a maximum in Tywi (Wales). The causes of different levels of introgression among river basins are unknown but might be related to either differing degrees of anthropogenic disturbance, mainly the construction of migration obstacles such dams and weirs [20,21], and/or innate differences relating to behavior or genetic composition. Whether natural or anthropogenically induced, changing environmental conditions have long been thought to play a major role in increasing rates of hybridization in fishes [45]. Several recent freshwater fish studies [46,47] underscore the importance of broader ecological degradation leading to increased hybridization rates, a situation which easily applies to the UK and Portuguese rivers where high levels of hybridization are documented. Nevertheless, shads represent a very unique case of introgressive hybridization among European fishes, something that needs to be further addressed using also nuclear markers.

### Geographic distribution of intraspecific genetic variability

Phylogeographic structure within *A. fallax* revealed by AMOVA and SAMOVA clearly relates to the major basins (Atlantic and Mediterranean), but also to smaller-scale patterns evidenced by regionally specific haplotypes. Therefore, it appears that differentiation was mainly shaped by historical processes of fragmentation between the Mediterranean and the Atlantic as described for many other species (e.g. species of the Sparidae family- marine [48,49]; brown trout- anadromous [4]), but also by regional barriers within these oceanographic regions. The divergence time estimates between the main clades marking the divide between the Mediterranean and the Atlantic *A. fallax* populations (0.20 to 0.30 Myrs between clade 2 and clade 1; Table 1) suggest that separation occurred after the Mindel glaciations, when a hypothetical ancestral population expanded its range through the three regions, allowing subsequent divergence. However, we do not know with certainty if fragmentation was caused by sea level drop during glacial maximums, shifting marine currents, or adaptation to different environmental conditions existing between the two basins. Thus, at least for *A. fallax,* the Strait of Gibraltar or a nearby region (e.g. the Almeria-Oran front) act in restricting gene flow between the Mediterranean and Atlantic, as described for many other species [17]. This barrier may not be absolute for *A. fallax*, considering the existence of shared haplotypes (Af3 and Af10) between the two basins and the coalescent-based migration estimates, with significant gene flow from the Mediterranean into the Atlantic (but not vice versa; Table 2). A simulation study testing the violations to isolation-with-migration models showed that gene flow with a third population, can inflate migration and effective population size estimates [44]. However, our estimates remained significant even when we corrected for this possibility (Additional file 1: Figures S1D, 1E and 1F), namely migration between Morocco and European Atlantic coast, suggesting that *A. fallax* migration from the Mediterranean into the Atlantic inferred using mtDNA is indeed real. Although migration over long distances through the Straits of Gibraltar is possible, we cannot exclude alternative possibilities associated with past connection between headwater captures of Mediterranean and Atlantic draining rivers, migration through artificial canals, or non-documented human-mediated transport related with stocking. Nonetheless, as no *A. fallax* haplotypes typical for the Atlantic region have been thus far found in the Mediterranean and *A. alosa* is currently limited to the Atlantic basin, it would appear as if the Atlantic-Mediterranean corridor is at least a contemporary isolating mechanism for these species.

The geographic origin of clade 3 in *A. fallax* is not clear. The fact that it reaches higher frequencies in the southern Atlantic populations from Morocco and Guadiana suggests an African or Southern Iberian origin. However, the lack of variability in the southern most population (Sebou) is puzzling. Huge population declines and possible local extinction has been suggested for *A. fallax* in Morocco [50], which could have resulted in a depletion of variability on that region. However, further studies using nuclear markers and samples from other African populations (if available) are needed to evaluate this hypothesis and to elucidate about the putative origin of clade 3.

At a smaller geographic scale, significant population structure (Table 6) supported by the existence of fixed or regionally restricted haplotypes is observed in *A. fallax* (Table 4). This pattern is especially evident in the Mediterranean, where the existence of relatively frequent haplotypes restricted to some regions (Af9 in Greece and Turkey; and Af19 in Corsica and Sardinia; Table 4) explain the higher differentiation found in this oceanographic region compared with the Atlantic (Table 6). This can either suggest a higher fidelity in terms of homing behavior of the *A. fallax* populations inhabiting the Mediterranean or a longer history of isolation accompanied by a more demographic stability of these populations. However, this is difficult to evaluate with the present data set, as the system of rivers draining to each of these oceanographic regions is not easily comparable.

Genetic variation of *Alosa* in the Atlantic basin shows both concordance between the two species, with respect to the haplotype networks, as well as strong differences in overall diversity and phylogeographic structure. In both species, three similarly divergent clades are present, most likely reflecting evolution in the same refugia during glacial maxima. Despite the fact that *A. alosa* displays little phylogeographic structure compared to *A. fallax* (Table 6), clades as a whole are broadly spread throughout the Atlantic basin in both species, where they co-exist (Figure 3). This pattern implies a concordant history of both species presumably due to the same climatic events and the same long-term refugia, yet differing in post-glacial demography and/or ecological responses to climatic amelioration (see below). *A. alosa* has a considerably more limited distribution and spawns much higher in the river systems than *A. fallax*, an obviously more demanding ecological niche, through periods of natural hydrological instability and glacial advance as well as in more contemporary times due to numerous anthropogenically caused interruptions in river corridors. Consequently, the task of inferring the geographic origin of the three main clades observed for *A. alosa* is comparatively much more challenging, as the species disappeared from some of the putative candidate regions (Mediterranean and Morocco). Future studies should thus make use of existing material (ancient DNA) and of *A. alosa* haplotypes “available” in populations of *A. fallax* through introgression, prior the local extinction of *A. alosa*, to identify the geographic origin of the clades identified here.

### Demographic responses to Pleistocene climate

Interestingly, although the two species share parallel intraspecific clade structures supporting parallel refugia, mtDNA haplotype diversity of *A. alosa* is much lower than for *A. fallax*. The rapidly declining range of *A. alosa*, including its disappearance from the Mediterranean basin and Northern Africa can explain the differences found between the two species [20,21], and have perhaps permanently clouded any trace of its evolutionary origins in geographic terms.

The demographic responses detected in our analysis were largely lineage specific, most likely reflecting relatively large-scale environmental changes. Assuming a neutral or nearly-neutral evolution of the mtDNA molecule, strong statistical support for post-glacial growth was seen for both *A. alosa* and *A. fallax* lineages in the Atlantic basin, with roughly similar age estimates, presumably reflecting expansion after the LGM (Table 6). Reproductive migrations of *A. fallax* are thought to be inhibited by water temperatures below 11°C [51] implying that this species must have been purged from, or at least drastically reduced in the North Atlantic during the LGM. However, not only present, but also historical effective population sizes of *A. fallax* may have been larger than for *A. alosa*. This may suggest that even before anthropogenically caused environmental changes *A. alosa* had a more limited capacity to survive climatic oscillations. However, we cannot exclude that a population of *A. alosa* may have survived in the North Atlantic, where haplotypes of clade 2 are more frequent (Figure 3B), a hypothesis that needs to be tested in the future with nuclear data. Regardless of the refugia location, it appears that the refugial populations of *A. alosa* have only carried limited mtDNA haplotype diversity at our level of resolution compared to *A. fallax*, which seems to have experienced more stable conditions along the Iberian coast compared to the North Atlantic.

Demographic growth in *A. fallax* (as suggested by Fu’s *F*_s_) seems to have started earlier in the Mediterranean than in the Atlantic (Table 7; Additional file 2: Figure S2), suggesting either a faster amelioration or the maintenance of adequate environmental conditions in the Mediterranean during the last glacial period (Würm), allowing the permanence of stable populations of *A. fallax* in the region. The actual rarity of *Alosa* on the southern shores of the Mediterranean probably reflects that the present day sub-tropical temperatures are far from optimal for this species. In general, for both *A. alosa* and *A. fallax*, the contemporary southern limit of their distributions appears to reflect marginal habitat conditions, due to warm temperatures. During the last century, the occurrence of *A. alosa* along the Mediterranean was mainly reported for the coast of the Iberian Peninsula and France, where *A. fallax* is presently abundant. The fact that this region exhibits the coolest water temperatures along the entire Mediterranean basin [52] further supports our hypothesis.

The distribution of the genetic diversity of European *Alosa* species throughout the Atlantic, in particular of *A. fallax* (clade 1), suggests that a southern refuge existed along the Iberian Peninsula (or further south), similar to that reported for many other species (e.g. marine- the common goby, *Pomatoschistus microps*[53]; freshwater- saramugo, *Anaecypris hispanica*[54]; anadromous- brown trout, *Salmo trutta*[55]; but see [56] for a review). However, contrary to what has been suggested for other anadromous fishes with Atlantic distribution, namely the cold-tolerant species Atlantic salmon *S. salar*[57] and brown trout *S. trutta*[55] (see also [58] for marine species) for which glacial refugia in Northern Europe have been suggested, *Alosa* northern populations seem to have been directly affected by the advance of ice sheets, as suggested by the low nucleotide diversity observed in these populations, when only the haplotypes of the clade with a putative Atlantic origin (clade 1) are considered. Thus, *Alosa*’s comparative cold-intolerance may limit the potential range of glacial refugia in the Atlantic, which, together with the fact that contemporary climatic conditions appear to be causing problems for the species at the southern limit of their distribution, underscores, especially for *A. alosa*, the sensitivity of these species to climatic change. In contrast to the Atlantic, the Mediterranean seems to provide more long-term stable habitat for lineages of *Alosa* during colder periods, but the opposite may also be true during interglacials, as suggested by the recent disappearance of *A. alosa* and the distribution of *A. fallax* throughout the region, with lower abundance in areas with higher seawater temperature.

## Conclusions

The geographically extensive sampling implemented in this study allowed us to shed light on the present day and historical distribution of Eurasian shad’s genetic diversity, providing a necessary framework upon which different evolutionary hypotheses can be further tested. *Alosa alosa* and *A. fallax* mtDNA haplotypes from different geographic locations cluster in two major lineages that roughly correspond to the two species, suggesting a unique event of diversification and subsequent colonization of different river basins. The observation of gene flow between these two species, which differs in intensity and directionality among geographic locations, suggest that the genomic architecture underlying pre- and/or post-zygotic barriers may differ geographically. Although we cannot neglect that these inferences were drawn based on a single locus, they provide fundamental information for new research projects. Further studies on Eurasian shads should focus on multiple molecular markers and on particular ecological features, in order to evaluate the nature of putative reproductive barriers between these species in different river systems where they coexist. Moreover it will be important to identify genomic regions impermeable to gene flow in order to understand the building up of the genetic basis of reproductive isolation. The repeated contact between these lineages across different river systems offers a unique set of replicated experiments for evaluating reproductive barriers and to test speciation mechanisms.

The high population differentiation of *A. fallax* populations, especially in the Mediterranean suggest a complex evolutionary history but also constitute an interesting region to study the genetic basis of adaptation to high water temperatures and salinity. This implies that a conservation perspective to the taxonomic diversity in this region should be more sensitive to other potential population differences (genetic, morphological and ecological).

Despite the similar haplotype network topologies between *A. alosa* and *A. fallax*, suggesting a common intraspecific evolutionary history, their different demographic histories, present day distribution ranges, and spawning niche differentiation reflect different ecological needs and responses to natural or human-mediated climatic changes. Shads are clearly relatively vulnerable taxa due to their dependence on major river systems, which are some of the most heavily impacted habitats in Europe. Their responses to the remarkable flow and sea level changes experienced naturally in the past may become even more dramatic due to the additive effects of current human activities. The differences observed between these two species in terms of the genetic diversity must be considered by management actions to insure the maintenance of their declining populations.

## Methods

### Sample collection

**Figure 4:**
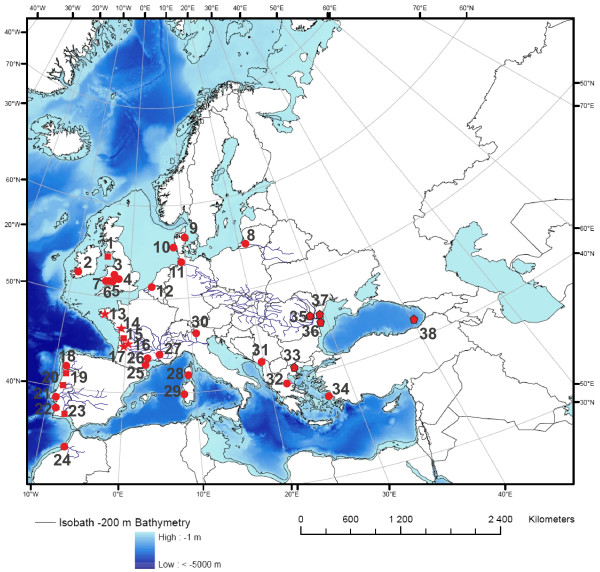
**Map of Europe showing the sampling locations.** Population key for *A. alosa* (red stars): 1- Scotland; 13- Aulne; 14- Vienne; 15- Charente; 16- Dordogne; 17- Garonne; 19- Lima; 20- Mondego; and 23- Guadiana. Population key for *A. fallax* (red circles): 1- Scotland; 2- Lake Leane, Ireland (landlocked); 3- Severn; 4- Teme; 5- Wye; 6- Usk; 7- Tywi; 8- Curonian Lagoon; 9- Ålbaek; 10- Wadden Sea; 11- Elbe; 12- Scheldt; 15- Charente; 18- Minho; 19- Lima; 20- Mondego; 21- Tejo; 22- Mira; 23- Guadiana; 24- Sebou; 25- Aude; 26 - Herault; 27- Rhone; 28- Corsica; 29- Sardinia; 30- Lake Garda (landlocked); 31- Lake Skadar (unknown migratory status); 32- Pinios; and 34- Izmir Bay. Population key for Black Sea/Lake Volvi species complex (red pentagons): 33- Lake Volvi (landlocked); 35- Tulcea; 36- St George; 37- Lake Isac; 38- Turkish Black Sea coast near Rice. Locations where both *A. alosa* and *A. fallax* were collected are marked with a red square; locations where only *A. alosa* was sampled are marked with a red star; locations where only *A. fallax* was sampled are marked with a red circle; locations where taxa from the Ponto-Caspio region and Lake Volvi were sampled are marked with a red pentagon. All populations are anadromous unless otherwise specified.

We sampled 29 populations of *A. fallax* and nine populations of *A. alosa* across their entire distribution range as well as individuals from the Black Sea/Lake Volvi region presumably representing *A. immaculata, A. caspia* and *A. macedonica*; and from the western Atlantic coast representing *A. sapidissima*, used as outgroup (Table 8). Individuals (*N* = 384) were captured by angling or nets between 1991 and 2003. In Figure 4, a map with the location of all sampling sites is provided (bathymetric data were extracted from the ETOPO2 dataset available on the US National Geophysical Data Centre (NGDC) [59]). Within *A. fallax*, five subspecies were analyzed (*A. f. fallax*, *A. f. killarnensis*, A*. f. lacustris*, *A. f. rhodanensis* and *A. f. nilotica*) (Table 8). For *A. alosa* and *A. fallax*, individuals were classified to species based on morphological appearance and whenever possible, based on a diagnostic morphological trait, the gill raker counts on the first left branchial arch, as described in Alexandrino et al. [28]; i.e. < 61 rakers = *A. fallax*, from 61 to 105 = hybrids, and > than 105 = *A. alosa*. A single exception was observed in Lake Garda, where *A. fallax* individuals have a higher number of gill rakers (between 56 and 64), as previously described [21]. Individuals identified as hybrids were excluded from further analyses. Tissue samples were preserved at −80°C or in 95% ethanol for molecular analysis.

**Table 8:** **List of individuals analyzed including their assigned taxa, basin, population code, country and sample location, including sample sizes (*N*) for each of the two genes characterized**

**Taxon**	**Basin**	**Pop. Code**^**1**^	**Country**	**Sample location**	***Cyt b*(*N*)**	*ND1***(*N*)**
*A. alosa*	Atlantic	1	Scotland	Solway firth	5	5
*A. alosa*	Atlantic	13	France	Aulne River	6	4
*A. alosa*	Atlantic	14	France	Vienne River	6	5
*A. alosa*	Atlantic	15	France	Charente River	7	7
*A. alosa*	Atlantic	16	France	Dordogne River	11	7
*A. alosa*	Atlantic	17	France	Garonne River	9	7
*A. alosa*	Atlantic	19	Portugal	Lima River	15	7
*A. alosa*	Atlantic	20	Portugal	Mondego River	7	5
*A. alosa*	Atlantic	23	Portugal	Guadiana River	9	8
*A. fallax fallax*	Atlantic	1	Scotland	Solway firth	13	5
*A. fallax killarnensis*	Atlantic	2	Ireland	Lake Leane	7	5
*A. fallax fallax*	Atlantic	3	U.K.	Severn River	10	5
*A. fallax fallax*	Atlantic	4	U.K.	Teme River	8	5
*A. fallax fallax*	Atlantic	5	U.K.	Wye River	14	6
*A. fallax fallax*	Atlantic	6	U.K.	Usk River	11	9
*A. fallax fallax*	Atlantic	7	U.K.	Tywi River	16	8
*A. fallax fallax*	Atlantic	8	Lithuania	Curonian Lagoon	7	5
*A. fallax fallax*	Atlantic	9	Denmark	Ålbaek	5	5
*A. fallax fallax*	Atlantic	10	Denmark	Wadden Sea	5	5
*A. fallax fallax*	Atlantic	11	Germany	Elbe River	5	5
*A. fallax fallax*	Atlantic	12	Belgium	Scheldt River	8	6
*A. fallax fallax*	Atlantic	15	France	Charente River	9	5
*A. fallax fallax*	Atlantic	18	Portugal	Minho River	7	7
*A. fallax fallax*	Atlantic	19	Portugal	Lima River	17	9
*A. fallax fallax*	Atlantic	20	Portugal	Mondego River	11	11
*A. fallax fallax*	Atlantic	21	Portugal	Tejo River	8	8
*A. fallax fallax*	Atlantic	22	Portugal	Mira River	10	5
*A. fallax fallax*	Atlantic	23	Portugal	Guadiana River	9	6
*A. fallax fallax*	Atlantic	24	Morocco	Sebou River	8	7
*A. fallax rhodanensis*	Mediterranean	25	France	Aude River	5	5
*A. fallax rhodanensis*	Mediterranean	26	France	Herault River	5	5
*A. fallax rhodanensis*	Mediterranean	27	France	Rhône River	7	6
*A. fallax rhodanensis*	Mediterranean	28	France	Corsica	6	6
*A. fallax rhodanensis*	Mediterranean	29	Italy	Sardinia	4	4
*A. fallax lacustris*	Mediterranean	30	Italy	Garda Lake	8	6
*A. fallax nilotica*	Mediterranean	31	Montenegro	Skadar Lake	5	5
*A. fallax nilotica*	Mediterranean	32	Greece	Pínios River	7	7
*A. fallax nilotica*	Mediterranean	34	Turkey	Izmir Bay	7	5
*A. macedonica*	Mediterranean	33	Greece	Volvi Lake	3	3
*A. caspia*	Black sea	37	Romania	Danube/Isac Lake	3	3
*A. immaculata*	Black sea	35	Romania	Danube/Tulcea	4	4
*A. immaculata*	Black sea	36	Romania	Danube/St. George	3	3
*A. immaculata*	Black sea	38	Turkey	Black Sea coast	5	5
TOTAL					335	249

^1^Population codes correspond to those in Figure 4.

### Molecular analysis

Whole genomic DNA was extracted using a standard high-salt protocol [60]. Genetic variation was screened at two mitochondrial DNA genes: a 515 bp fragment of *cyt b* and the complete *NADH-1* (*ND1*: 975 bp) by polymerase chain reaction (PCR) for 4–20 fish per population (Table 8). New data produced in this study were combined with 29 individuals analyzed in Faria et al. [25] and 75 *cyt b* sequences presented in Alexandrino et al. [28] (GenBank accession numbers DQ419761-DQ419775, DQ419782-DQ419802; and AY937212-AY937217, respectively).

Both genes were amplified using *Alosa* specific primers, described for *cyt b* (Alocytbf1 and Alocytbr1) in Alexandrino et al. [28] and for *ND1* (Alond1f1 and Alond1r1) in Faria et al. [25]. Amplification conditions for the *cyt b* are described in Alexandrino *et al.* (2006). *ND1* was amplified as follows: each 25 μl reaction contained 14.7 μl H_2_O, 2.5 μl of Ecotaq specific reaction buffer (Ecogen), 0.8 μl of each primer (10 mM), 3 μl and of 50 mM MgCl_2_, 1 μl of 10 mM dNTP’s, 0.2 μl Ecotaq *Taq* DNA polymerase (Ecogen), and approximately 100 ng of DNA template. The cycle parameters were as follows: initial denaturation at 94°C for 2 min; denaturation at 94°C (30 s), annealing at 62°C for *cyt b* and 63°C for *ND1* (30 s), and extension at 72°C (30 s) repeated for 35 cycles; and a final extension for 3 min at 72°C. PCR products were purified with ExoSap-IT (USB) and sequenced following the ABI PRISM BigDye Terminator Cycle Sequencing protocols, in one direction using either Alocytbf1 or Alocytbr1 for *cyt b*, and using two internal primers (Alond1f2 and Alond1r2; [25]) for the *ND1*. Sequencing products were electrophoresed on either an ABI PRISM 310 or 3100 Genetic Analyzer (PE Applied Biosystems). The sequences of the different haplotypes produced during this study are available in GenBank (accession numbers: JX080076-JX080177). Alignments were done separately for each gene using BioEdit 5.0.9 [61].

### Data analysis

#### Phylogenetic inference

Interspecific relationships were primarily evaluated through the construction of bi-furcating trees. Maximum Parsimony (MP), Maximum Likelihood (ML); Bayesian (B) and Neighbor-Joining (NJ) reconstruction methods were used for both genes concatenated. We used MODELTEST 3.06 PPC [62] under the Akaike information criterion (AIC) to obtain a most likely model of nucleotide substitution that was subsequently used in the B and ML inference, the latter carried out with the software PHYML 3.0.1 [63]. Maximum Parsimony (MP) analysis was carried out in PAUP (version 4.0b10, [64]) using a heuristic search and the tree bisection and reconnection (TBR) branch swapping method. The Bayesian analysis was implemented in MRBAYES v3.1.2 [65], using a Metropolis-coupled, Markov chain Monte Carlo (MCMC) sampling approach. The Bayesian analysis was carried out with two simultaneous runs starting by random trees. Each run consisted of four chains, with default heating parameters, and 10,000,000 generations sampled every 1,000 steps. The standard deviation of split frequencies was used as a convergence index (<0.01). Convergence as well as congruence across runs was further assessed with the online version of AWTY [66]. The first 25% samples were discarded as burn-in and the remaining summarized in a majority rule (50%) consensus tree, with nodes’ support expressed as posterior probabilities. The software MEGA (version 3.1, [67]) was used to perform NJ analysis based on Tamura-Nei distances. The robustness of all nodes was assessed by bootstrap analysis with 1,000 pseudoreplicates [68]. To test for rate homogeneity (i.e. the existence of a molecular clock), we compared the likelihood of the ML unconstrained tree with the likelihood of obtaining the same topology when enforcing a molecular clock, using a likelihood ratio test (LRT) [69]. MEGA was also used to calculate mean (*D*_*xy*_) and net nucleotide divergence (*D*_a_) between the haplogroups that correspond to the main taxonomic units here analyzed (*A. alosa*, *A. fallax* and BSC including Bs2) as well as between intraspecific lineages (within *A. alosa* and *A. fallax*) based on uncorrected *p*-distances. As there is no reliable calibration for mtDNA divergence rate in Alosinae, estimates are presented for a range of conventional rates (1-4%/Myrs), but discussions of phylogeographic scenarios are centered around an assumption of a conventional divergence rate of 2%/Myrs for the entire mtDNA [70].

#### Coalescent-based divergence time and migration

To test if shared haplotypes between *A. fallax* and *A. alosa* resulted from introgression or incomplete lineage sorting, we employed the isolation‐with‐migration model implemented in IMa2 software [71]. We considered a two-population model, estimating the six parameters involved: current and ancestral population sizes (θ1, θ2 and θA, respectively), effective number of migrants in each direction (2Nm1 and 2Nm2) and time since population split (*t*). The HKY model was used [72]. Prior bounds were first based on the authors’ recommendations and subsequently optimized. At least five independent runs of each data set were performed and effective sample size of parameters (ESS) and trend plots examined to assure proper mixing and convergence. The significance of migration rate estimates was measured by log-likelihood ratio tests (LLR) [73]. A nucleotide substitution rate of 1% (mtDNA) and an average generation time of five years were used to obtain biologically meaningful estimates. Additionally, we employed this same procedure to address the same questions for the main *A. fallax* geographic groups of populations (Atlantic and Mediterranean), assuming a generation time of four years. This was done both including and excluding haplotypes from clade 3, to understand if hypothetical gene flow with a third population would substantially change our estimates.

#### Phylogeographic analysis and summary statistics

Multi-furcating networks were constructed to evaluate intraspecific relationships among all haplotypes identified both in *A. alosa* and *A. fallax*, including the ones shared among species, using the Median-Joining (MJ) algorithm [74] implemented in the program NETWORK (version 4.1.1.2, [75]). A Reduced Median (RM) haplotype network, which according to the authors contains less superfluous links by resolving parallel mutations, was also estimated using NETWORK for each species individually (excluding the haplotypes shared between the two species). Among *A. fallax* sequences, three different nucleotides were observed in two of the entire set of variable positions, presenting a problem for RM algorithm, which only takes binary data. Because only two amendments were needed to transform the entire dataset into a binary format, we manually replaced the entry of these haplotypes in the input file, following the authors’ suggestions [75]. The corresponding mutations were subsequently manually added to the obtained network. Haplotype diversity (*h*), nucleotide diversity (*π*) and mutation parameter (*θ*), were calculated using the software DNASP (version 4.0, [76]). These parameters were calculated for each species separately, as well as for intraspecific clades.

#### Analysis of molecular variance and population structure

To evaluate the genetic variability within and among populations of *A. alosa* and *A. fallax* we performed a two-level hierarchical AMOVA [77] using ARLEQUIN software (version 3.01, [78]). For *A. alosa*, a two-level AMOVA was performed considering all the sampling sites, but also considering solely the northern Atlantic sampling sites (1,13–17), as well as the southern Atlantic sampling sites (19, 20, 23), separately. For *A. fallax*, a two-level AMOVA was performed considering all the sampling sites, but also solely for the sampling sites located in the Atlantic (1 to 24), as well as the ones located in the Mediterranean (25 to 34, except 33), separately.

Additionally, to test if a major geographic barrier to gene flow for *A. fallax* exists among European Atlantic, Mediterranean and Moroccan coasts, populations were grouped in three main regions (sampling sites 1 to 23 - Atlantic; sampling sites 25 to 33 - Mediterranean; and sampling site 24 - Morocco), and a three-level hierarchical AMOVA was performed. The SAMOVA software (Spatial Analysis of Molecular Variance; version 1.1, [79]) with the number of groups (*k*) set to three and a default number of simulated annealing procedures (100) was further used to test if the grouping we chose for the AMOVA analysis was the one that maximized the proportion of total genetic variance due to differences among groups, when *k* = 3. Population structure analyses were performed after excluding putative introgressed individuals.

#### Demographic history

To elucidate events of population expansion and decline, we used several neutrality tests, namely, Tajima's *D*[80], Fu’s *F*_*S*_[81] and *R*^2^ statistics [82], which have also been applied in drawing demographic inferences, assuming the neutrality of the mtDNA genes here analyzed. DNASP was also used to calculate these statistics and their significance using 10,000 coalescent simulations for each data set. When population growth was detected, we also estimated θ_0_ = 2*N*_0_μ (before population expansion), θ_1_ = 2*N*_1_μ (after expansion) and the age of expansion (τ = 2μ*t*, where μ is the substitution rate per Myrs, and *t* is the time in Myrs), assuming a one-time sudden exponential growth model.

In addition, Bayesian skyride analyses [83] implemented in BEASTv.1.7.1 [84] were performed to explore demographic change through time within each clade of *A. fallax* and *A. alosa,* except for those composed by only two haplotypes (clade 3; and clade 2 and 3, respectively). However, as conclusions that can be drawn based on these powerful model-based coalescent approaches applied to a single locus are very limited [85], the results here presented are merely suggestive. The Bayesian skyride, which performs a temporal smoothing of the effective population size, was used as tree prior by choosing the time-aware smoothing procedure [83], as it seems to perform better than other approaches implemented in BEAST (e.g. Bayesian skyline plot) under a wide range of demographic scenarios [83]. The appropriate model of nucleotide substitution was estimated for each clade based on the concatenated dataset using jMODELTESTv0.1.1 [86] following the Bayesian information criterion (BIC). For each clade, the model suggested by jMODELTEST was used under a lognormal relaxed molecular clock (assuming a relaxed substitution rate of 1%) [87], and using an UPGMA-based (unweighted pair group method with arithmetic mean) starting tree. Runs of 30–120 million steps were performed and results visualized using TRACERv1.5 [88]. Three independent runs were performed for each data set to check for convergence using TRACER. The demographic analyses were performed per clade, under the assumption that in the past each clade formed single panmictic population. As this assumption may often be unrealistic, the results of the demographic analyses are presented as a comparison among clades and should not be interpreted in terms of their absolute values. All the demographic analyses were performed after excluding putative introgressed individuals.

## Competing interests

The authors declare no competing interests of any kind.

## Authors’ contributions

All authors participated in the experimental design and development of this research study. PA was the scientific coordinator of the research projects that supported this study. RF carried out the molecular and statistical analysis within the framework of his PhD studies. RF wrote the first draft of manuscript, which was substantially improved by SW. All authors read, revised and approved the final version.

## Authors’ information

RF is a post-doc fellow with a diverse interest in evolutionary biology and is currently involved in the study of chromosomal speciation. SW is an associate professor specialized in the ecology, evolution and conservation management of freshwater fishes, especially salmonids. PA is an associate professor and the head of the Evolutionary Ecology and Genetics of Aquatic Organisms research group at CIBIO, with special interest on the study of the evolution and conservation of aquatic organisms and with a broad experience on the study of shads.

## Supplementary Material

Additional file 1 **Figure S1.** This file contains figures supposed to be displayed as supplementary material. In these figures are presented the posterior probability distribution curves of the splitting times, effective population sizes and migration estimates using IMa2. Click here for file

Additional file 2 **Figure S2.** This file contains figures that are supposed to be displayed as supplementary material. In these figures are presented the Bayesian skyride plots for the *A. alosa* and *A. fallax* clades for which changes in effective population size through time were detected. Click here for file
