# Supplementary material for: Comparative phylogeography and demographic history of European shads (Alosa alosa and A. fallax) inferred from mitochondrial DNA
Source: BMC Evol Biol. 2012 Sep 30;12:194. doi: 10.1186/1471-2148-12-194 (PMC3523006; doi:10.1186/1471-2148-12-194)

**Figure 1. Probability distributions based on IMa2.** A) Splitting time between *A. fallax* and *A. alosa*; B) effective population size of *A. fallax*, *A. alosa* and of their ancestral population; C) effective number of migrant gene copies per generation between *A. alosa* and *A. fallax*; D) Splitting time between the Mediterranean and Atlantic Populations of *A. fallax*; E) effective population size of Mediterranean and the Atlantic *A. fallax* populations and of their the ancestral population; F) effective number of migrant gene copies per generation between Mediterranean and Atlantic *A. fallax* populations. From D to F, two probability distributions are shown for each parameter: 1) Including all sequences; 2) excluding sequences putatively originated from a third group (Morocco).

**A**


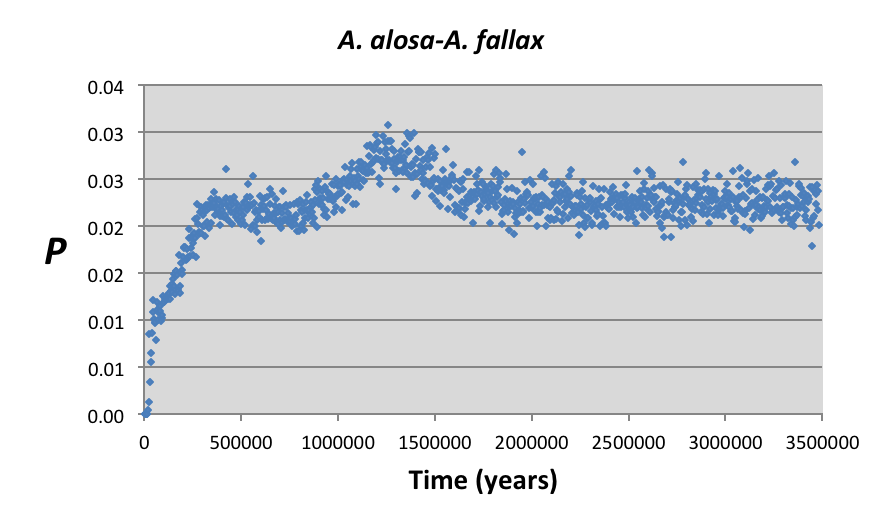


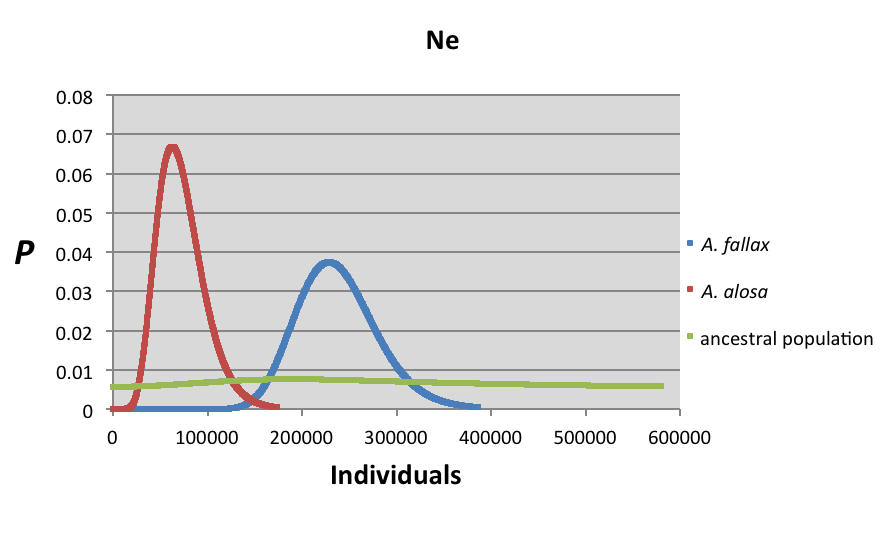


**B**


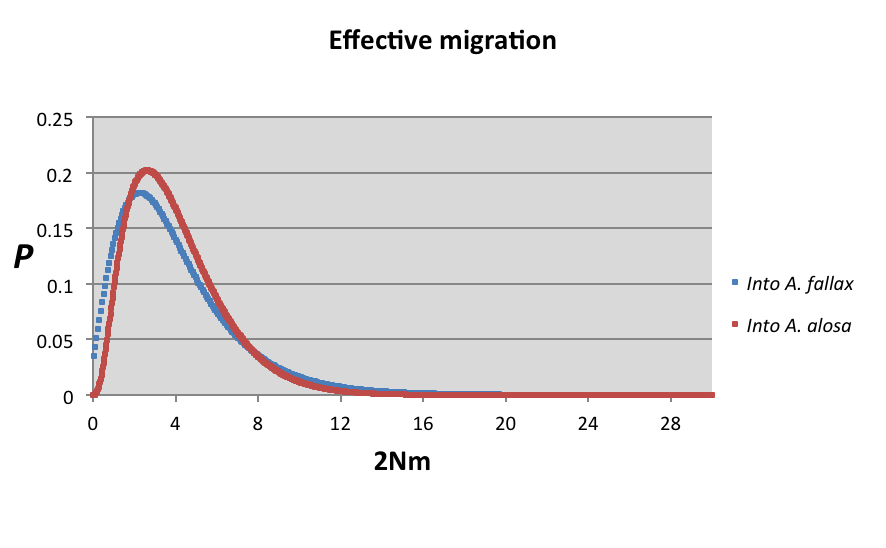


**C**


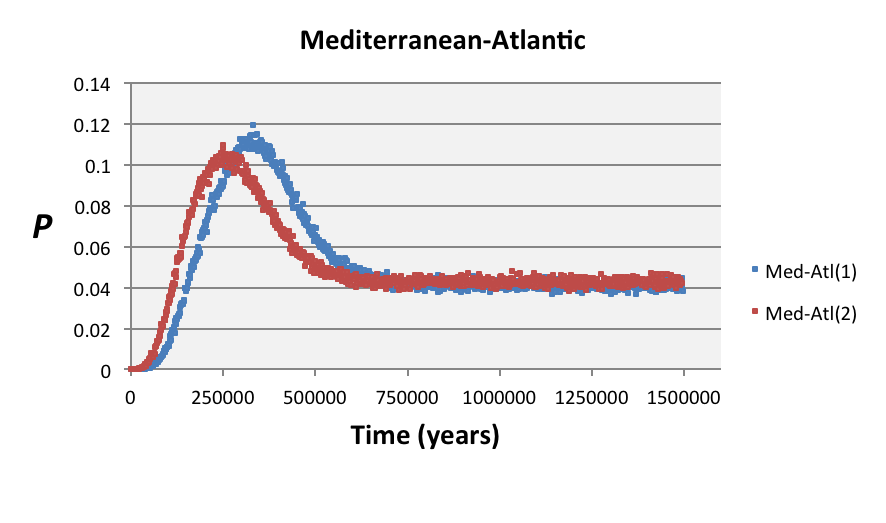


**D**


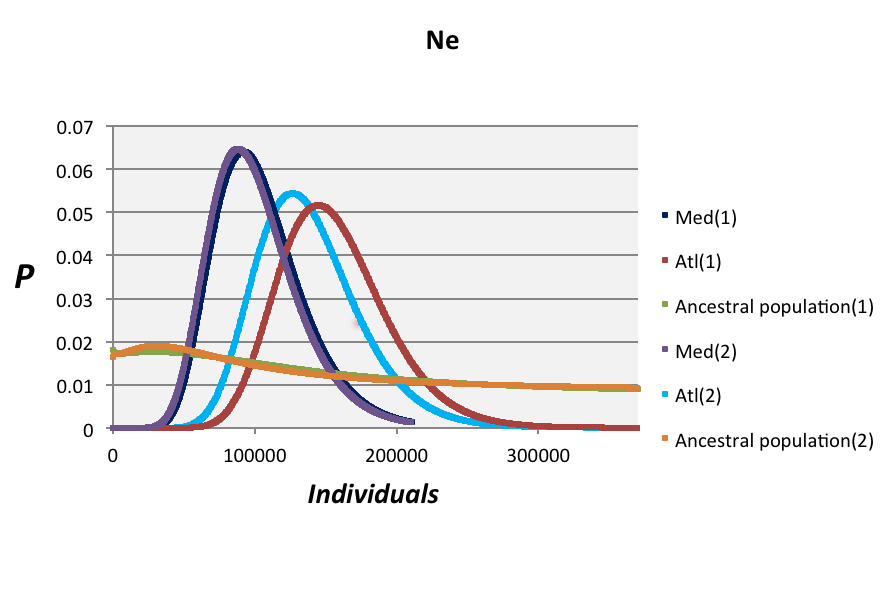


**E**

**F**


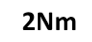

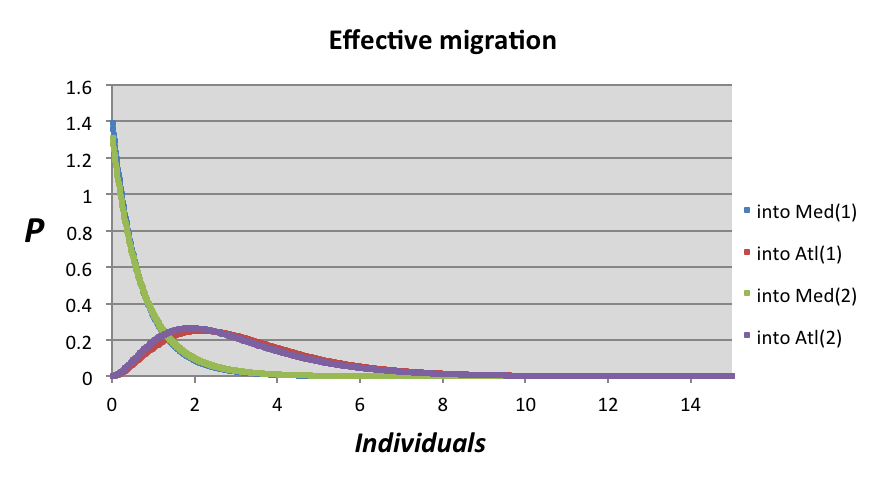

Supplement: Additional file 1 — Figure S1. This file contains figures supposed to be displayed as supplementary material. In these figures are presented the posterior probability distribution curves of the splitting times, effective population sizes and migration estimates using IMa2. [file 1471-2148-12-194-S1.doc]
