# Supplementary material for: Comparative phylogeography and demographic history of European shads (Alosa alosa and A. fallax) inferred from mitochondrial DNA
Source: BMC Evol Biol. 2012 Sep 30;12:194. doi: 10.1186/1471-2148-12-194 (PMC3523006; doi:10.1186/1471-2148-12-194)

**Figure 2. Bayesian skyride plots of different mtDNA clades of European shads.** Shown are the changes in effective population size through time (in years before the present). Solid lines represent median bayesian skyride plot values, while blue lines represent the 95% highest probability density (HPD) intervals. The maximum time (X-axis) represents the root height’s median, while dashed vertical lines represent the root height’s lower 95% HPD. The y-axis values are scaled by mutation rate and are presented on a logarithmic scale. (A) clade 1 of *A. fallax*; (B) clade 2 of *A. fallax*; (3) clade 1 of *A. alosa*.

**A**


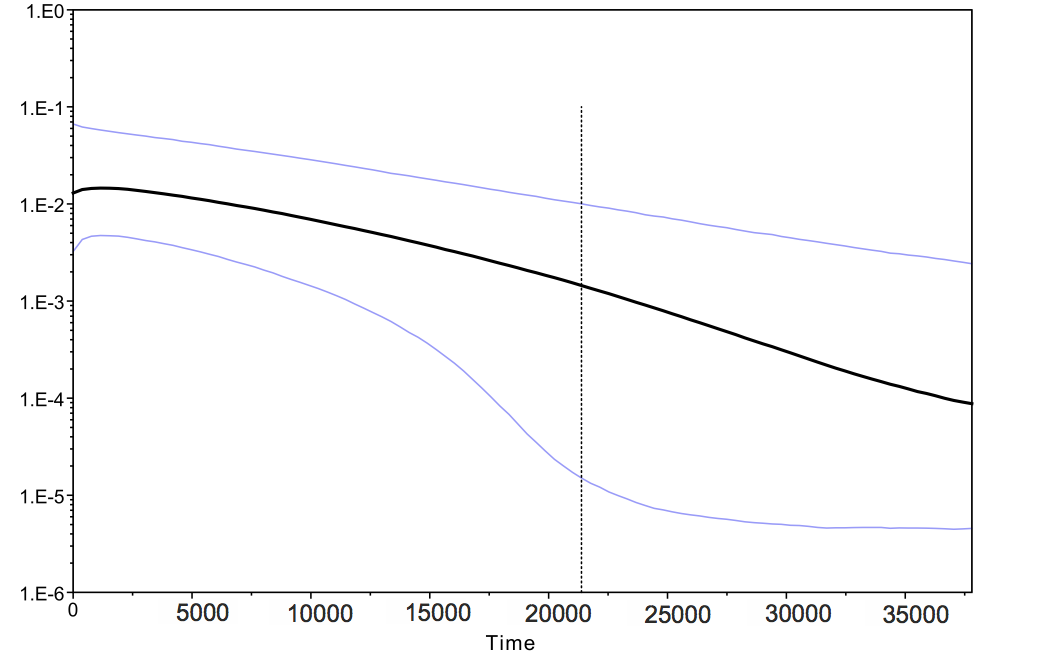


**B**


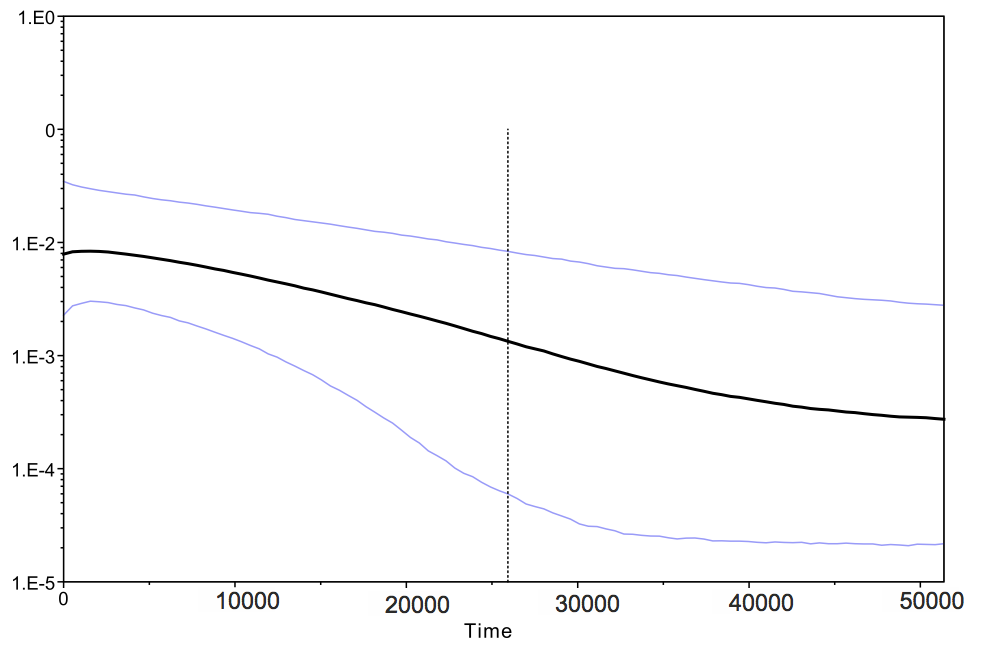


**C**


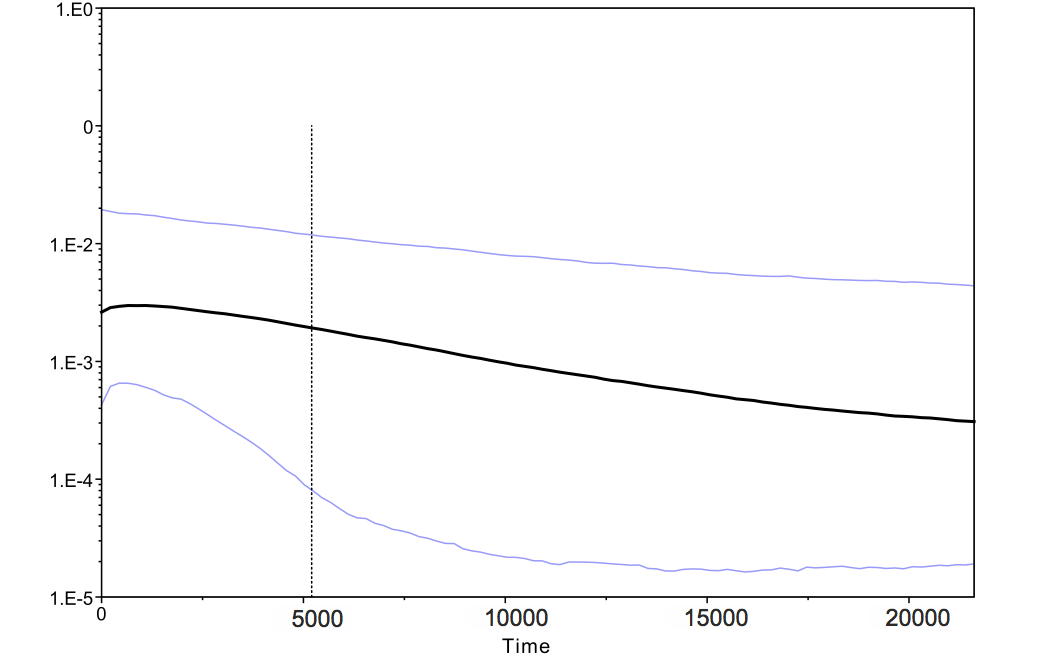

Supplement: Additional file 2 — Figure S2. This file contains figures that are supposed to be displayed as supplementary material. In these figures are presented the Bayesian skyride plots for the A. alosa and A. fallax clades for which changes in effective population size through time were detected. [file 1471-2148-12-194-S2.doc]
